# Editorial for the Special Issue “Genetics Studies on Wheat”

**DOI:** 10.3390/genes14091761

**Published:** 2023-09-04

**Authors:** Hongxiang Ma

**Affiliations:** Joint International Research Laboratory of Agriculture and Agri-Product Safety, The Ministry of Education of China/Jiangsu Key Laboratory of Crop Genomics and Molecular Breeding/Jiangsu Co-Innovation Center of Modern Production Technology of Grain Crops, Yangzhou University, Yangzhou 225009, China; mahx@yzu.edu.cn

## Editorial

Wheat (*Triticum aestivum* L.) is one of the most widely cultivated cereal crops, contributing approximately one-fifth of the total calories consumed by humans and provides more protein than any other food source [1]. Breeding for improved cultivated varieties by tuning genetically complex yield and end-use quality parameters while maintaining yield stability and regional adaptation to specific biotic and abiotic stresses is necessary to promote the sustainable development of the wheat industry and ensure worldwide food security and safety [2]. Genetic studies are the basis of wheat breeding; however, the insufficient knowledge and understanding of the genetic mechanism and molecular basis of key agronomic traits has limited breeding efforts. Therefore, the research on wheat genetics and breeding technology needs to be strengthened to improve the accuracy and efficiency of wheat breeding. In this context, this Special Issue, “Genetics Studies on Wheat”, presents recent research relating to quantitative trait loci (QTL) identification, gene mining, molecular characterization, and genetic improvement of multiple traits in wheat.

Most traits, including yield potential, grain quality, and the resistance to biotic and abiotic stress, are known as quantitative traits influenced by the environment and controlled by QTL [3]. Usually, the identification of QTL and the development of markers associated with such traits via molecular mapping with specific genetic populations is the first step in marker-assisted selection and gene mining. QTL identification based on biparental mapping requires the construction of segregating populations by crossing parental lines; obtaining stable traits is time-consuming. The genome-wide association study (GWAS), based on the linkage disequilibrium (LD) between polymorphic molecular markers and the causal gene, is currently a mainstream method for mining prediction genes. GWAS overcomes the limitations of biparental QTL, shortens breeding time, reduces energy cost, and provides high-resolution mapping, allowing researchers to accurately delineate the prediction region to accelerate the crop-breeding process [4]. The GWAS approach depends on large panels of breeding lines or genotypes collected from naturally evolved and adapted populations with wider genetic variation. Often, with this approach, researchers can identify smaller intervals using historical recombination events with polymorphic markers, such as single-nucleotide polymorphisms (SNPs). As a result, dense maps and high statistical mapping resolution facilitate the identification of SNPs associated with the studied trait [5,6,7]. These associations may provide key markers for trait introgression, marker-assisted selection, or targets for functional manipulation for crop improvement. Using this approach, the resistances to wheat blast and spot blotch, grain metal accumulation, and hardiness are reported in this Special Issue. Phuke et al., (2022) found that 2NS translocation was the only major source and explained up to 32% of the phenotypic variation in the resistance to wheat blast via a GWAS of 7554 filtered SNP markers and 350 Indian wheat genotypes in 12 field experiments in 3 different locations. Additional marker-trait associations were located on chromosomes 2B, 5A, and 7A [8]. Lozano-Ramirez et al., (2022) evaluated a collection of 441 synthetic hexaploid wheat lines for resistance to spot blotch and identified 41 significant marker–trait associations, located on chromosomes 1B, 1D, 2A, 2B, 2D, 3A, 3B, 3D, 4A, 4D, 5A, 5D, 6D, 7A, and 7D [9]. El-Soda and Aljabri screened 200 advanced lines of spring wheat from the wheat association mapping initiative for Mn, Fe, Cu, Zn, Ni, and Cd concentrations. Positive correlations were found between all metals except Ni and Cd. Of a total 142 significant SNPs, 26 showed possible pleiotropic effects on 2 or more metals [10]. GWAS was also used to identify the association loci associated with the grain hardness index. A total of five significant association loci were identified; four of them, distributed on chromosomes 1A and 7D, could be detected in three or more environments [11].

For gene identification and molecular characterization, Guo et al., (2022) combined the results of QTL mapping with a set of 184 recombinant inbred lines and the GWAS of 272 varieties for wheat plant height and identified a novel reduced-height gene encoding ATP-dependent DNA helicase (*TaDHL-7B*). The plant height significantly decreased compared with that of the wild type when *TaDHL-7B* was knocked out using the CRISPR/Cas9 system in the ‘Fielder’ variety. This is the first reduced-height gene with a nonhormone pathway isolated in wheat [12]. To improve phosphorus use efficiency in wheat, Abbas et al., (2022) identified 22 wheat orthologs of the *phosphorus starvation tolerance 1* (*PSTOL1*) gene, which was reported to play a key role in efficient P uptake, deeper rooting, and high yield in rice. In the study, they found that wheat *PSTOL1* orthologs are unevenly distributed on chromosomes, some of them are colocalized with phosphorus-starvation-related QTLs, and wheat *PSTOL1* genes showed differential expression patterns in different tissues under different phosphorus regimes. These results strengthen the classification of Pakistan-13 as a P-efficient cultivar and Shafaq-06 as a P-inefficient cultivar. The results of phenotypic characterization demonstrated that P-efficient cultivar Pakistan-13 has significantly higher P uptake, longer root length, larger root volume, and greater root surface area compared with P-inefficient cultivar Shafaq-06 [13].

Wheat contains three subgenomes (A, B, and D), with total genome size of approximately 16 Gb, 80% of which is highly repetitive DNA sequences [14]. Due to a high genome complexity, extensive pan-genome studies in common wheat lag compared with those in other crops such as rice, maize, and soybean. Zheng and Zhang (2022) reanalyzed the genome sequences from 16 different wheat varieties and identified 62,299 core genes. The core genes could be classified into genes related to tissue development and stress responses according to their expression profiles, including 3376 genes highly expressed in both spikelets and at high temperatures. After associating with six histone markers and open chromatin, they found that these core genes could be divided into eight subclusters with distinct epigenomic features; 51% of the expressed transcription factors were marked with both H3K27me3 and H3K4me3, which are involved in tissue development through the transcription factor centered regulatory network [15].

Considerable achievements in wheat breeding have ensured the food security worldwide in recent decades. The selection of high yield potential in wheat breeding has narrowed the genetic background of cultivated varieties; most varieties cannot cope with biotic and abiotic stress, which seriously affects wheat development. Therefore, determining the genetic diversity of landraces can provide highly valuable information that will help us to broaden the genetic base of the germplasm used in breeding programs. Ethiopia is considered a center of origin and diversity for durum wheat, being endowed with many diverse landraces. In a study, 104 durum wheat varieties originating from Ethiopian wheat genotypes were investigated for their genetic diversity using 10 grain-quality- and grain-yield-related phenotypic traits and 14 simple sequence repeat makers. The results of the phenotypic-data-based principal component analysis, the molecular-data-based discriminant analysis of the principal components, and minimum spanning network analyses defined distinct groupings of cultivars and landraces. The phenotypic and molecular diversity analyses highlighted the high genetic variation in the Ethiopian durum wheat gene pool. The investigated SSRs showed significant associations with one or more target phenotypic traits. The markers identified landraces with high grain yield and quality traits. This study highlights the usefulness of Ethiopian landraces for cultivar development [16].

Related wild species are important for broadening the genetic diversity of wheat and retain many characteristics that common wheat lacks through natural evolution, such as disease resistance, insect resistance, and drought tolerance. Dai et al., (2022) used a trigeneric hybrid, YZU21, with resistance to FHB, powdery mildew, and stripe rust, to improve two major wheat cultivars, Ningmai 13 and Yangmai 23, in the middle to lower reaches of the Yangtze River in China. Five addition or substitution lines and one translocation line of the *Triticum–Secale–Thinopyrum* trigeneric hybrid were obtained by using specific molecular markers and GISH. The agronomic trait evaluation and the resistance to multiple diseases showed that the six trigeneric hybrid lines have desirable agronomic traits and improved resistance to FHB, powdery mildew, and stripe rust [17].

Two reviews are also included in this Special Issue. Wan et al., (2003) reviewed a breeding strategy for the application of synthetic hexaploid wheat (SHW)—the large population with limited backcrossing method. The stripe-rust-resistance and big-spike-related QTLs/genes from SHW were pyramided into new high-yield cultivars with this method. SHW represents an important genetic basis of big-spike wheat in southwestern China. Furthermore, multispike and preharvest sprouting resistance QTLs/genes from other germplasms were pyramided to SHW-derived cultivars based on phenotypic and genotypic evaluations by using a recombinant inbred line-based breeding method. Consequently, the improved wheat varieties created the highest yield record in southwestern China [18].

Chang et al., (2023) reviewed the genetic improvement of wheat with preharvest sprouting (PHS) resistance in China. The research progress of germplasm evaluation and use, QTL identification, gene function, and the molecular mechanisms of the resistance to PHS were summarized in this context. Different breeding methods have been applied to enhane the resistance to PHS, most wheat varieties with high resistance to PHS were bred via the conventional hybridization method under strict selection pressure. Molecular marker-assisted selection was effectively used to improve the efficiency and accuracy in breeding for resistance to PHS, creating wheat varieties such as Zhongmai 911 and Annong 0711. Genetic transformation and gene editing were also studied to improve the resistance to PHS; the results showed application potential, but no breeding varieties have been released for wheat production [19].

In summary, this Special Issue collates a selection of papers broadly covering the field of genetics in wheat breeding. The manuscripts focus on QTL/gene identification, marker development, gene function, epigenetics, and related wild species application for grain nutrition, phosphorus use efficiency, plant height, and resistance to wheat diseases and preharvest sprouting, with a strong interest in wheat breeding. The study results provide a solid foundation for wheat genetics improvement. Although conventional breeding techniques are still indispensable in wheat breeding at present, marker-assisted selection breeding has been increasingly applied, and transgenic and gene editing techniques have been studied, showing considerable application potential. This Special Issue provides a valuable resource for improving our knowledge of the genetic bases of important traits and breeding strategy in wheat.
